# Clinical course, radiological findings and late outcome in preterm infant with suspected vertical transmission born to a mother with severe COVID-19 pneumonia: a case report

**DOI:** 10.1186/s13256-021-02835-0

**Published:** 2021-04-23

**Authors:** Roya Farhadi, Shahrokh Mehrpisheh, Vajiheh Ghaffari, Mohammadreza Haghshenas, Aghdas Ebadi

**Affiliations:** 1 Pediatrics infectious diseases research center, Mazandaran University of Medical Sciences, Sari, Iran; 2 Molecular and Cell Biology Research Center, Faculty of Medicine, Mazandaran University of Medical Sciences, Sari, Iran; 3 Department of Obstetrics and Gynecology, Mazandaran University of Medical Sciences, Sari, Iran

**Keywords:** COVID-19, SARS-CoV-2, Neonate, Preterm infant, Vertical transmission

## Abstract

**Background:**

Vertical transmission of coronavirus disease 2019 (COVID-19) from mother to newborn infant is doubtful, and very little is known about disease severity and neonatal outcome.

**Case presentation:**

We present a preterm Iranian infant born to a Persian mother with severe COVID-19 pneumonia. The mother underwent cesarean delivery, and amniotic fluid yielded a positive result for severe acute respiratory syndrome coronavirus 2 (SARS-CoV-2) by real-time reverse transcription polymerase chain reaction (RT-PCR). The newborn infant showed early-onset infection with SARS-CoV-2 confirmed on pharyngeal swabs by RT-PCR assay within 24 hours after birth, suggesting vertical transmission. Unfortunately, the mother died 14 days after delivery. We describe the clinical course and outcome of the infant up to 7 months of age.

**Conclusion:**

COVID-19 infection in pregnant women may increase maternal morbidity, mortality and possibly vertical transmission in severe cases. However, it does not seem to progress to serious early or late neonatal complications.

## Background

Pneumonia caused by the novel severe acute respiratory syndrome coronavirus 2 (SARS-CoV-2) was reported in Wuhan, China in December 2019 [1, 2]. On February 11, 2020, the World Health Organization (WHO) officially named the disease caused by the novel virus, coronavirus disease 2019 (COVID-19) [3, 4]. The outbreak was declared a pandemic by WHO as the virus spread rapidly to other countries including Japan, Korea, Thailand and Iran, with high mortality rates [3, 5]. It seems that due to physiological and immunological changes during pregnancy, pregnant women are susceptible to COVID-19 and more likely to have morbidity and mortality [1, 6]. At present, there is not enough data to ascertain the effects of COVID-19 infection on the fetus, and the main question is whether vertical transmission of COVID-19 from pregnant mother to fetus occurs and causes clinically significant infection in newborns [5, 7, 8]. Indeed, published case series and case reports have shown extensive variability in the rate of vertical transmission. As COVID-19 infection can potentially cause severe complications in neonates, continuous observance is necessary [9]. On the other hand, the disease severity when SARS-CoV-2 is transmitted vertically and the time of this transmission are still uncertain. Thus, WHO recently published a definition and categorization system for the timing and possibility of infection occurrence to enhance our understanding of SARS-CoV-2 vertical transmission and its clinical outcomes [10].

In this report, we present early-onset infection with SARS-CoV-2 in a preterm infant, born to a mother with severe COVID-19 pneumonia. The mother’s clinical characteristics have already been published in a research letter [11]. In this article we focus more on the clinical course and para-clinical evidence for the newborn, which indicates the possibility of vertical transmission of SARS-CoV-2 from mother to infant, and we describe the follow-up of the infant to 7 months of age.

## Case presentation

On March 11, 2020, the neonatal resuscitation team of Imam Khomeini Hospital in Sari (northern Iran) was alerted for the delivery by caesarean section of a mother with critical COVID-19 pneumonia. The mother was a 22-year-old, primigravid Persian woman suffering from fever, nonproductive cough, myalgia, anorexia and nausea for the preceding two weeks. She was a housewife and came from a middle-class family and had a history of exposure to a person with COVID-19 infection 1 week before the onset of symptoms. She did not smoke or drink alcohol. Prior to admission, she had received only oral azithromycin and acetaminophen. Diagnosis of COVID-19 pneumonia was confirmed by lung computed tomography (CT) scan and two positive nasal and throat sample real-time reverse transcription polymerase chain reaction (RT-PCR) tests (SuperScript III Platinum, Quantitative Real-Time PCR kit, Invitrogen, Waltham, MA, USA), and she underwent antiviral treatment and respiratory support. During admission, treatment with Kaletra (lopinavir/ritonavir 200 mg orally daily), azithromycin (500 mg intravenously every 12 hours), ceftriaxone (1000 mg every 12 hours), Tamiflu (oseltamivir orally, 400 mg every 12 hours) and hydroxychloroquine (400 mg daily) were initiated. The mother did not receive antenatal corticosteroid, and considering the rapid deterioration of her condition and the recommendation of a multidisciplinary working group, a decision was taken for an early caesarean delivery at 32 weeks of gestation.

Neonatal resuscitation team members wore the recommended personal protective equipment (PPE) and followed the COVID-19 protocols for perinatal care. The mother only had a history of hypothyroidism without other underlying diseases such as diabetes. Hypothyroidism was controlled by treatment with levothyroxine 100 μg daily. Following the policy during the early first wave of COVID-19, we decided in advance to separate the baby from the mother immediately after birth and postpone skin-to-skin contact and delayed cord clamping.

Five milliliters of amniotic fluid was aspirated into a sterile syringe with a needle after myometrial incision of the uterus, just before tearing of the membrane, for COVID-19 RT-PCR assay. A preterm female Persian infant was delivered uneventfully. She needed only initial steps of resuscitation and had APGAR scores of 8 and 9 at 1 and 5 minutes, respectively. Umbilical cord blood samples were obtained in sterile containers with viral transport media to immediate transfer to the specific virology center of the university for COVID-19 studies.

**Fig. 1:**
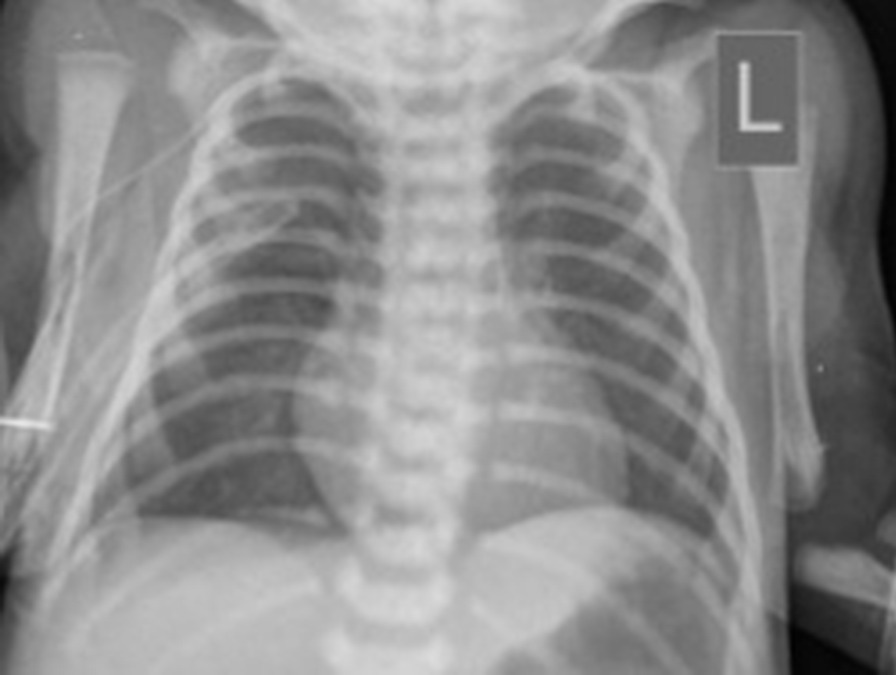
Chest radiograph of the newborn obtained on the fifth day of life

**Fig. 2:**
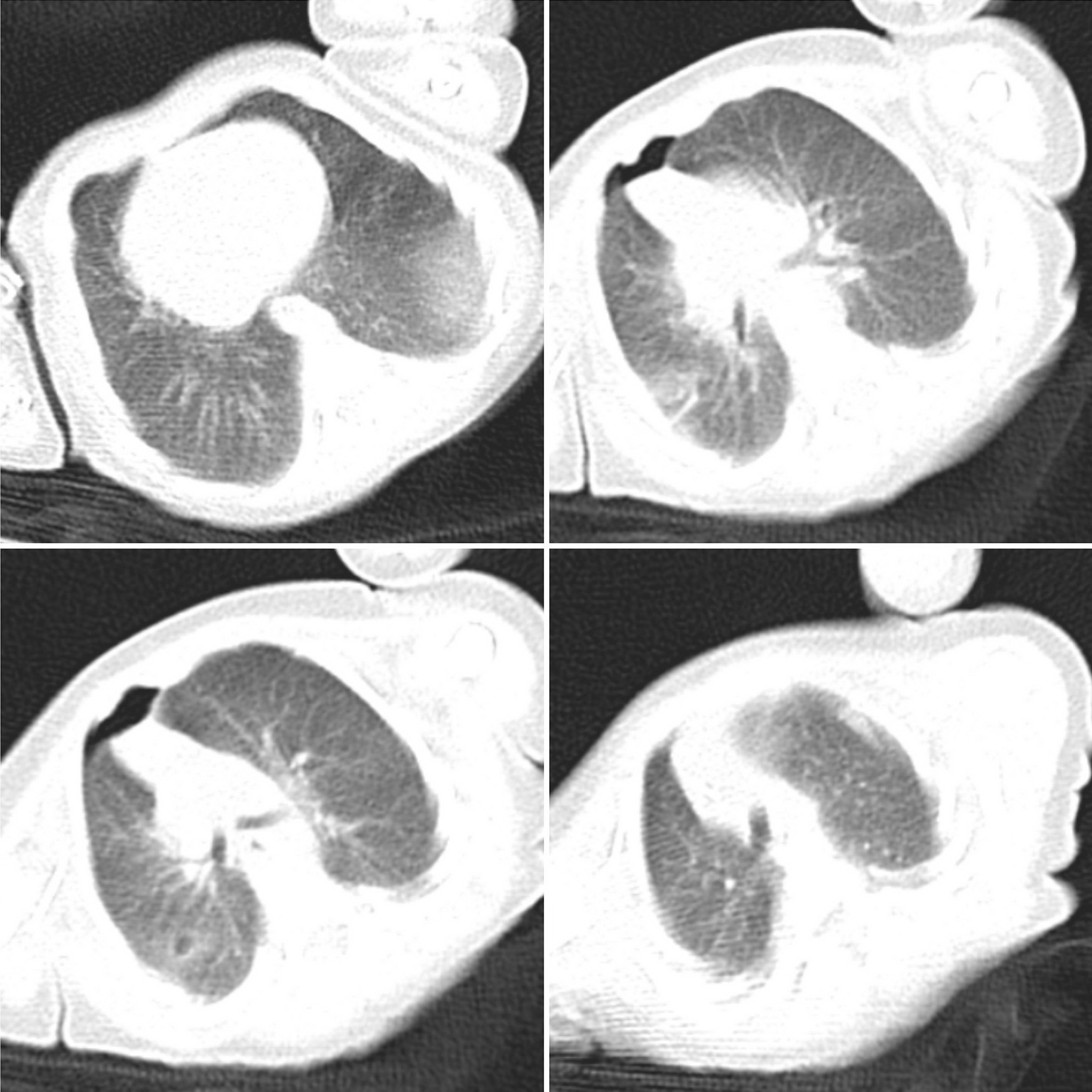
Chest computed tomography of the baby obtained on the ninth day of life showing bilateral patchy shadows

The newborn infant weighing 2350 gm was isolated in a single-occupancy room with an air filtration system in the neonatal intensive care unit (NICU). She had respiratory distress and required continuous positive airway pressure (CPAP) with positive end-expiratory pressure (PEEP) of 5 cmH_2_O and fraction of inspired oxygen (FiO_2_) of 21%. The first chest X-ray showed characteristic features of respiratory distress syndrome (RDS). According to the updated protocols, neonatal nasopharyngeal and oropharyngeal swab samples were collected at 2 and 24 hours after delivery and tested for SARS-CoV-2 using the SuperScript III Platinum Quantitative Real-Time PCR kit (Invitrogen, Waltham, MA, USA) at the medical virology laboratory of Mazandaran University of Medical Sciences. Twelve hours after birth, the infant was formula-fed because the mother was critically ill, and we did not have a milk bank in our hospital. Intravenously administered ampicillin (50 mg/kg/dose every 12 hours)  and gentamicin (4 mg/kg/dose every 48 hours)  were prescribed as empirical antibiotic therapy. Noninvasive respiratory support (nasal CPAP) was required for 2 days, with acceptable blood gas analysis. Umbilical cord and first oro/nasopharyngeal sample RT-PCR tests reported negative results; however, the results of amniotic fluid and the second pharyngeal swab done within 24 hours turned positive for SARS-CoV-2. The neonate’s blood gas analysis was acceptable, and blood oxygen saturation stayed above 92% without supplemental oxygen. Other laboratory results including complete blood count, C-reactive protein, creatine phosphokinase, lactate dehydrogenase, aspartate aminotransferase, alanine aminotransferase, thyroid-stimulating hormone and alkaline phosphatase were within the normal range. Two days after discontinuing the CPAP, while the neonate was afebrile, the baby became unwell with respiratory distress and increasing need for supplemental oxygen. The antibiotic regimen was changed to vancomycin (10 mg/kg/dose every 18 hours) and meropenem (20 mg/kg/dose every 12 hours). Chest X-ray showed a pneumomediastinum/pneumothorax needing chest drain insertion due to oxygen desaturation (Fig. 1). Subsequent to the chest drain removal, she remained stable on 30% supplemental nasal cannula oxygen, and a lung CT scan revealed patchy shadows in the peripheral parts of the lung (Fig. 2).

The infant was not commenced on specific antiviral treatments. On day 14 of life (March 24), a repeat pharyngeal swab sample was obtained, and the result was positive. The results of repeated pharyngeal swab samples for SARS-CoV-2 on the 16th and 18th days of life were negative. When the baby's general condition improved, we decided to share the baby's pictures and videos with the mother in the ICU. This decision was made based on a quality improvement project in our unit [12]. We did not succeed because the mother underwent peritoneal dialysis, intubation and full sedation for ventilator therapy due to oxygen desaturation. Unfortunately, the mother died on March 26 due to the severity of her illness.

The neonate received a total of 14 days of antibiotics and was discharged home on day 30 of life. She was followed up with mobile communication and telemedicine clinics initially and subsequently through limited clinic visits. Eye examinations for retinopathy of prematurity were normal. Hearing screening test results showed clear response for both of the baby’s ears as well. The results of ultrasound of the brain and lungs performed at 2 months of age were normal. Neurodevelopmental status was appropriate for the corrected gestational age when reviewed in late October.

## Discussion

In this report we present the case of a preterm baby, born to a mother with severe COVID-19 pneumonia. The newborn infant had positive RT-PCR tests on the first and 14th days of life and pulmonary involvement on lung CT scan. Although findings from a recent small group of cases indicate that there is currently no strong evidence for vertical transmission in women who develop COVID-19 pneumonia late in pregnancy, the possibility of maternal-to-fetal vertical transmission cannot be completely ruled out, given the small sample size reported, and poses a concern among neonatal societies [13, 14]. According to the WHO classification, the reasons suggesting vertical transmission in our neonate are the positive amniotic fluid test and positive RT-PCR testing of the baby within 24 hours of life [10]. In addition, the baby had respiratory distress, although it is difficult to differentiate COVID-19 symptoms from the symptoms of RDS, transient tachypnea of the newborn (TTN) and sepsis [14].

The possibility of vertical transmission of COVID-19 infection from mother to baby has already been proposed. Alzamora *et al*. reported a preterm infant born to a mother with severe COVID-19 from Peru. The infant’s nasopharyngeal swab at 16 hours after birth was positive for SARS-CoV-2 on RT-PCR. Immunoglobulins (IgM and IgG) for SARS-CoV-2 were negative. The authors did not test for the presence of the virus in the amniotic fluid. Similar to our case, they believe that the severity of maternal illness could be a possible explanation for vertical transmission [15]. Another report from India also highlighted the possibility of vertical transmission of COVID-19 from a mildly symptomatic mother [16].

In a report of nine pregnant women with laboratory-confirmed COVID-19 pneumonia in Wuhan, China, cord blood, amniotic fluid and neonatal throat swab were tested for the presence of SARS-CoV-2 and all samples were negative for the virus. Four mothers had preterm labor, but all were beyond 36 gestational weeks. The authors concluded that development of COVID-19 pneumonia in the third trimester could not lead to fetal infection or severe adverse outcome in neonates by intrauterine vertical transmission [13].

Dong *et al*. reported possible vertical transmission in a newborn with elevated IgM antibodies to SARS-CoV-2 born to a mother with COVID-19 in China. The baby was exposed for 23 days from the time of the mother’s diagnosis of COVID-19 to delivery, but the infant’s repeated RT-PCR nasopharyngeal swabs tests were reportedly negative, and PCR testing of amniotic fluid was not done [17]. Due to the limited number of confirmed neonatal cases, there is still a lack of clarity as to whether the duration of maternal infection affects the rate of positive test results in neonates [14]. On the other hand, the viral load and amount of virus may be another important factor for amplification of RT-PCR test results and transmission to infants [18, 19]. In our case, the mother exhibited severe pneumonia and subsequently died. It is possible that when the mother exhibits a severe disease course with the associated presence of the virus in the amniotic fluid, the vertical transmission potential also increases, probably due to the high viral load. In a cohort study, Zeng *et al.* recruited all 33 neonates born to mothers with COVID-19 from Wuhan Children’s Hospital in China. Of the three newborns who tested positive for COVID-19 infection, the first RT-PCR test for all three babies was taken on the second day of life. Therefore, the authors were unable to determine whether these babies caught the virus in the womb or were infected by contact transmission around the time of birth [20]. In our study, the first RT-PCR test was negative and the second was positive, raising the question of prenatal or postnatal timing of infection. On the other hand, the amniotic fluid test was positive, and the baby was born by cesarean section with our strict measures to reduce the risk of infection. We believe that it is unlikely that the infant was infected during the delivery.

At present, RT-PCR is considered the gold standard for the diagnosis of SARS-CoV-2 [21]. However, the positive detection rate of the nasopharyngeal swab test is reportedly less than 50% [7, 8].

The scope to which maternal COVID-19 infection increases the risk of preterm delivery remains uncertain, though based on several unpublished reports from Spain, prematurity was secondary to an obstetrics decision to terminate the pregnancy due to the severity of the disease in the mother [21]. In our case report, preterm delivery was due to severe pneumonia and respiratory insufficiency in the mother.

The limitation of our study is that we were not able to measure the neonatal SARS-CoV-2 IgM level because the serologic kits were not available to us in the early stages of the pandemic.

## Conclusion

The case reported herein of an infected preterm neonate born to a mother with critical infection suggests that COVID-19 infection in pregnant woman may increase the risk of maternal mortality, and vertical transmission may be possible in severe cases. Therefore, the collection of additional evidence regarding perinatal infection is of significant interest for assessing the risk of vertical transmission of SARS-CoV-2 and its impact on neonatal outcomes.

## Data Availability

All data and material collected during this study are available from the corresponding author upon reasonable request.

## References

[1] Wang S, Guo L, Chen L (2020). A case report of neonatal 2019 coronavirus disease in China. Clin Infect Dis..

[2] Yu N, Li W, Kang Q (2020). Clinical features and obstetric and neonatal outcomes of pregnant patients with COVID-19 in Wuhan, China: a retrospective, single-centre, descriptive study. Lancet Infect Dis..

[3] Karimi-Zarchi M, Neamatzadeh H, Dastgheib SA (2020). Vertical transmission of coronavirus disease 19 (COVID-19) from infected pregnant mothers to neonates: a review. Fetal Pediatr Pathol..

[4] Hui DS, Azhar IE, Madani TA (2020). The continuing 2019-nCoV epidemic threat of novel coronaviruses to global health—the latest 2019 novel coronavirus outbreak in Wuhan, China. Int J Infect Dis..

[5] Mimouni F, Lakshminrusimha S, Pearlman SA, Raju T, Gallagher PG, Mendlovic J (2020). Perinatal aspects on the covid-19 pandemic: a practical resource for perinatal-neonatal specialists. J Perinatol..

[6] Mardani M, Pourkaveh B (2020). A controversial debate: vertical transmission of COVID-19 in pregnancy. Arch Clin Infect Dis..

[7] Lu Q, Shi Y (2020). Coronavirus disease (COVID-19) and neonate: what neonatologist need to know. J Med Virol..

[8] Chen Y, Peng H, Wang L (2020). Infants born to mothers with a new coronavirus (COVID-19). Front Pediatr..

[9] Goh XL, Low YF, Ng CH, Amin Z, Ng YPM (2021). Incidence of SARS-CoV-2 vertical transmission: a meta-analysis. Arch Dis Child Fetal Neonatal Ed..

[10] World Health Organization: Definition and categorization of the timing of mother-to-child transmission of SARS-CoV-2. 8 February 2021. Geneva: World Health Organization; 2021.

[11] Zamaniyan M, Ebadi A, Aghajanpoor S, Rahmani Z, Haghshenas M, Azizi S (2020). Preterm delivery, maternal death, and vertical transmission in a pregnant woman with COVID-19 infection. Prenat Diagn..

[12] Farhadi R, Mehrpisheh S, Philip RK (2021). Mobile-assisted virtual bonding enables breast milk supply in critically Ill mothers With COVID-19: a reflection on the feasibility of telelactation. Cureus.

[13] Chen H, Guo J, Wang C (2020). Clinical characteristics and intrauterine vertical transmission potential of COVID-19 infection in nine pregnant women: a retrospective review of medical records. Lancet.

[14] Ma X, Zhu J, Du L (2020). Neonatal management during the coronavirus disease (COVID-19) outbreak: the Chinese experience. NeoReviews.

[15] Alzamora MC, Paredes T, Caceres D, Webb CM, Valdez LM, La Rosa M (2020). Severe COVID-19 during pregnancy and possible vertical transmission. Am J Perinatol..

[16] Kulkarni R, Rajput U, Dawre R (2020). Early-onset symptomatic neonatal COVID-19 infection with high probability of vertical transmission. Infection.

[17] Dong L, Tian J, He S (2020). Possible vertical transmission of SARS-CoV-2 from an infected mother to her newborn. JAMA.

[18] Qiu L, Liu X, Xiao M (2020). SARS-CoV-2 is not detectable in the vaginal fluid of women with severe COVID-19 infection. Clin Infect Dis..

[19] Fan C, Lei D, Fang C (2021). Perinatal transmission of COVID-19 associated Sars-CoV-2: should we worry?. Clin Infect Dis..

[20] Zeng L, Xia S, Yuan W, *et al*. Neonatal early-onset infection with SARS-CoV-2 in 33 neonates born to mothers with COVID-19 in Wuhan, China (published online ahead of print, 2020 Mar 26). JAMA Pediatr. 2020; e200878. 10.1001/jamapediatrics.2020.0878 PMC7099530 32215598

[21] Chandrasekharan P, Vento M, Trevisanuto D (2020). Neonatal resuscitation and postresuscitation care of infants born to mothers with suspected or confirmed SARS-CoV-2 infection. Am J Perinatol.

